# Neuronal effects of epicranial current stimulation in macaque cortex

**DOI:** 10.3389/fnins.2025.1627705

**Published:** 2025-09-23

**Authors:** Boateng Asamoah, Ahmad Khatoun, Maria C. Romero, Elsie Premereur, Peter Janssen, Myles Mc Laughlin

**Affiliations:** ^1^Department of Neurosciences, ExpORL, KU Leuven and the Leuven Brain Institute, Leuven, Belgium; ^2^Laboratory for Neuro- and Psychophysiology, Department of Neurosciences, KU Leuven and the Leuven Brain Institute, Leuven, Belgium

**Keywords:** epicranial stimulation, transcranial electric stimulation, functional magnetic resonance imaging, entrainment, 40 Hz stimulation

## Abstract

**Background:**

Transcranial electrical stimulation (TES) using scalp electrodes is noninvasive, safe and inexpensive. However, because the scalp shunts most of the current, electric fields (E-fields) in the brain are relatively weak. Conversely, invasive neuromodulation methods such as deep brain stimulation (DBS) and invasive cortical stimulation (ICS) successfully treat many brain diseases. However, the expensive and risky surgery limits the reach of these approaches. Epicranial current stimulation (ECS), where electrodes are implanted on the skull, is a novel approach which can bridge the gap between these two extremes. In current study we investigated the effects of ECS on neural activity.

**Methods:**

In two macaque monkeys we implanted two concentric ring electrodes directly on the skull. Each electrode targeted one area PFG (PFG is not an acronym; rather it is the full name of a particular part of the parietal cortex) of the parietal convexity. Furthermore, a craniotomy was drilled in the skull to access the same area PFG. While recording (2 min) we stimulated (during the second recording minute) with a 10 or 40 Hz sinewave using an unfocused montage (between two electrodes on each side of the head) or a focused (through the concentric electrodes) over an intensity range of 0.25 to 4 mA. These two montages allowed us to investigate neural responses to targeted and broad brain stimulation. Furthermore, in a functional magnetic resonance imaging (fMRI) experiment we stimulated, at only 10 Hz, through an unfocused montage.

**Results:**

Our results show that E-field strengths depended on a combination of montage and stimulation intensity. Depending on the montage stimulation caused entrainment as well as spike rate increases. For focused stimulation and unfocused stimulation at lower amplitudes neural activity became entrained to the stimulation (similar to TES). For the unfocused stimulation, as stimulation amplitude increased, spike-rates also increased (similar to ICS and DBS) while the unfocused did not affect spike rates. The fMRI study showed a distributed pattern of activations which is suggestive of a network response caused by ECS.

**Conclusion:**

ECS has been used as a proxy for transcutaneous stimulation in rodent setups. Here we show that as a standalone technique it can be applied to a larger and more complex brain. This makes it a promising neuromodulation approach with clinical applications in patients who do not respond to TES but are not yet candidates for ICS or DBS.

## Highlights

- Epicranial stimulation as a sustained approach to brain stimulation.
- Epicranial stimulation causes widespread and robust activations in the brain.
- Large electric-field range generated in the brain with a montage-intensity interplay.
- Middle way between cortical stimulation and transcranial electric stimulation.

## Introduction

Invasive neuromodulation methods such as deep brain stimulation (DBS) (Gardner, 2013) and invasive cortical stimulation (ICS) (Penfield and Boldrey, 1937) have become accepted therapies and research tools in brain disorders such as essential tremor and epilepsy (Benabid, 2003; Della Flora et al., 2010; Kar and Krekelberg, 2012; Penfield and Boldrey, 1937; Wu et al., 2020). Recent decades have witnessed a renaissance of noninvasive brain stimulation (NIBS) methods such as transcranial electric stimulation (TES) (Asamoah et al., 2019; Khatoun et al., 2019a; Krause et al., 2019; Ozen et al., 2010; Vöröslakos et al., 2018) where a small current is applied to the scalp. Its non-invasive nature makes it an ideal tool for cognitive neuroscience and many studies have demonstrated its performance in healthly and diseased brains (Brittain et al., 2013; Kar and Krekelberg, 2014; Marshall et al., 2006; Mellin et al., 2018; Riecke et al., 2018; Santarnecchi et al., 2016).

Nevertheless, TES effects are often weak and difficult to reproduce (Héroux et al., 2017). This is partly due to scalp shunting which makes brain electric fields (E-fields) weak. Notably, some studies showed that the shunted current in the scalp is strong enough to activate cranial and cervical nerves (Asamoah et al., 2019; Tyler et al., 2015; Vanneste et al., 2020) and have observed TES neuromodulation can largely be traced back to this peripheral activation. Although current is also shown to directly affect neural activity (Asamoah et al., 2019; Kasten et al., 2019; Vöröslakos et al., 2018).

Epicranial current stimulation (ECS) is a neuromodulation method where electrodes are implanted under the scalp, directly on the skull (Khatoun et al., 2021). It has been employed to study stimulation and surgical techniques. However, as a standalone approach to long duration neuromodulation it is novel. In a computational study we showed that ECS E-fields are approximately one order of magnitude stronger than TES E-fields (Khatoun et al., 2019b). In the same study we furthermore showed that insulating electrodes prevents shunting between implanted electrodes thereby effectively hindering stimulation of skin nerves. As such, ECS stimulation intensities can be increased to relatively higher amplitudes. However, this comes at the cost of more invasiveness making it a better fit as potential treatment for neurological disorders rather than cognitive research. A currently running clinical trial has already shown some benefits of this stimulation approach (Kravalis and Schulze-Bonhage, 2020; Schulze-Bonhage et al., 2023). Despite its clinical use we know very little about its effects on neurons and the brain at large. Increased understanding will allow us to better design devices and stimulation waveforms and protocols to target varying brain regions and diseases. This would increase the potential groups of patients who may benefit from it. To investigate this gap in the knowledge, we implanted two Macaque monkeys with ECS electrodes and studied the effects of focused and unfocused montages using extracellular action potential recordings and functional magnetic resonance imaging (fMRI) during 10 and 40 Hz sinewave stimulation. We selected these frequencies due to the benefits of 10 Hz stimulation shown in the motor, auditory and visual systems (Garcia-Rill et al., 2016; He et al., 2022; Moliadze et al., 2019; Wach et al., 2013). Furthermore, mounting evidence show 40 Hz stimulation is beneficial in an array of brain functions (Hainke et al., 2025; Kong et al., 2025; Singer et al., 2018). The focused and unfocused montages allowed us to investigate neural responses to targeted and brain wide stimulation. We found that ECS produced relatively strong E-fields which could cause neural entrainment as well as spike-rate increases. fMRI analysis showed ECS to activate the target region in addition to a group of other cortical areas.

## Methods

### Subjects and surgery

**Figure 1:**
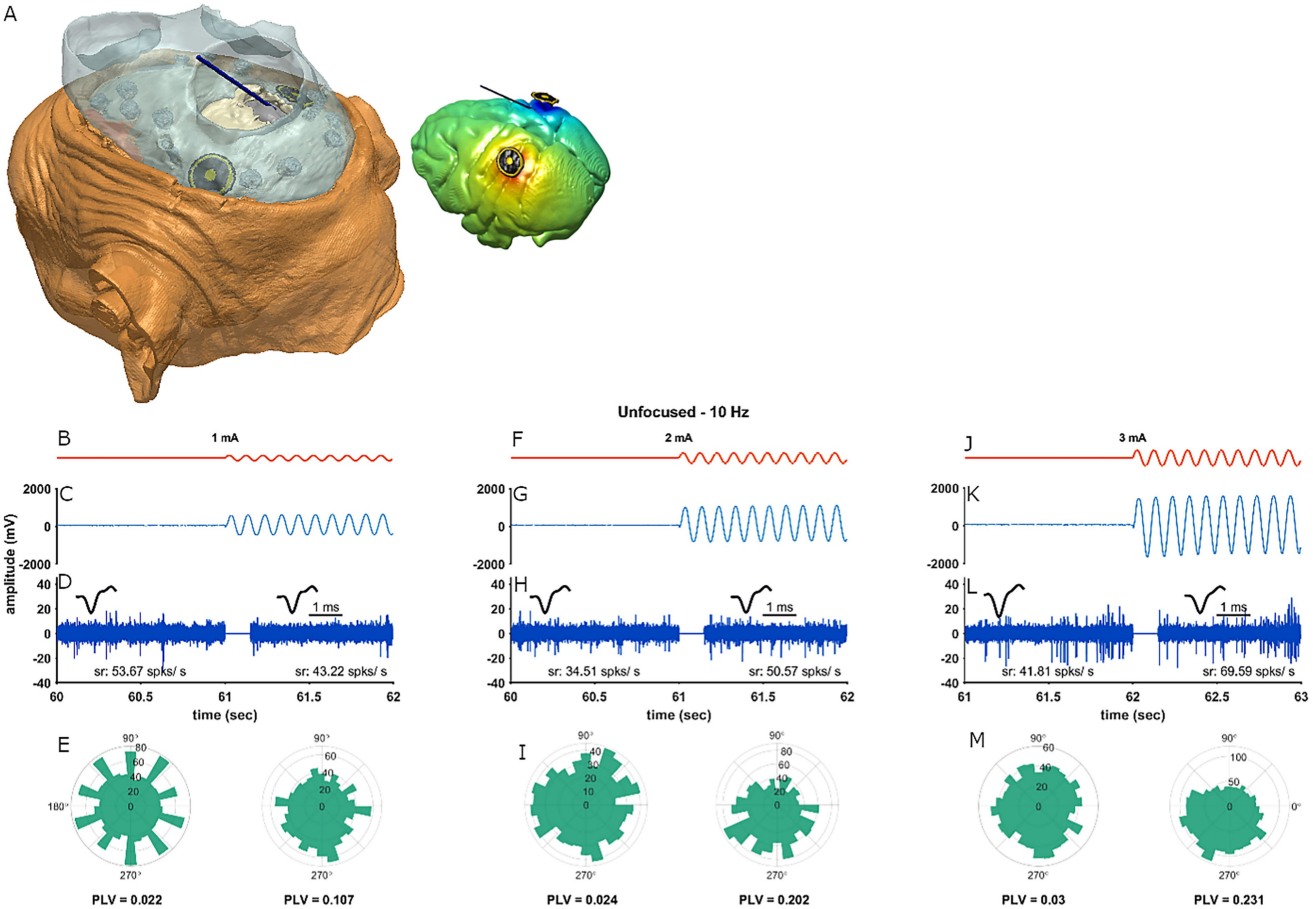
Experimental setup and protocol. **(A)** Electro-anatomical head model of monkey P from a left-dorsal birds-view perspective based on a CT scan. The dental cement is opaque and in blue; it shows a view on the skull (white) and the craniotomy which in turn gives access to the brain (grey). An electrode (dark blue line) penetrates the brain through the recording chamber and the craniotomy. The concentric ring stimulation electrodes (yellow shows the actual electrode contacts and black shows the insulated separation between the contacts) were placed such that the parietal convexity of area PFG could be targeted. The inset shows the same model but only with the recording and stimulating electrodes as well as the brain which is modeled with the generated electric potentials from 1 mA unfocused stimulation. **(B–E)** Stimulation and recording protocol. **(B)** Depicts the stimulation protocol of an example recording in orange (stimulation-OFF condition followed by a stimulation-ON condition at 1 mA). **(C)** Shows the recorded neural signal (blue) during the last second before and first second after stimulation onset. We shortened this depiction for reasons of legibility. In **D** the neural signal was filtered between 300 and 3,000 Hz for spikes. The insets above and below the filtered neural signal show average spikes and spike rates, respectively. After extracting OFF- and ON-condition spike times separately, cycle histograms in relation to the stimulation phase were calculated (**E**, polar histograms). From this we calculated entrainment (PLV, below the polar histograms) for the two stimulation conditions. The polar histograms and the PLV’s are based on the full recording. **(F–M)** Show data from the same recording but at 2 and 3 mA, respectively. Notice that this same recording site is depicted in Supplementary Figure 1 and entrains to a different phase.

Two male Rhesus monkeys (*Macaca mulatta*) were trained to sit in a primate chair. Under strict sterile conditions and propofol anesthesia (10 mg/kg/h), they then underwent two surgeries each. During the first surgery a headpost (Crist Instruments) was implanted on the skull and fixed using ceramic screws and dental cement which effectively functioned as an insulating layer to prevent current flow through the skin (see Figure 1A). At least six weeks later, both animals were trained in a passive fixation task. For this task they received a juice reward for fixating at a point on a screen in front of them. The task did not serve any experimental purpose; rather, it was to keep the monkeys alert during neural recordings. During the second surgery, a craniotomy above area PFG on the parietal convexity was drilled (Figure 1A) above the right hemisphere for monkey P and above the left hemisphere for monkey D, and a recording chamber was implanted over the craniotomy at approximately a 45° angle in relation to the mid sagittal line. This placement allowed an oblique entry into the parietal convexity we recorded only from this hemisphere. In the same surgery, a concentric ring electrode (CRE medical, Kingston, United States, outer ring diameter: 10 mm, center disk diameter 2 mm) was implanted directly on the skull to target the same PFG area. A second concentric ring electrode (same dimensions) was implanted on the other side of the head to target the same brain region on that side. This montage allowed for focused as well as unfocused stimulation, both described below.

Each of the 2 Monkeys was housed in an enclosure (3x3x2.5 m W x L x H) – with two other monkeys – which contained swings and perches and bedding on the floor. The layout of the enclosure was changed weekly and monkeys received toys which were scattered around. They had daily contact with researchers and care takers. Around experiment days they received a total of 1 Liter of liquid (water and juice reward); otherwise their liquid intake was not restricted. All procedures adhered to the National Institutes of Health guide for the care and use of laboratory animal, the EU directive 2010/63/EU and were also approved by the animal ethics committee for laboratory experiments at KU Leuven (ethics approval number: P126/2017).

### Electrophysiology: action potential and electric field acquisition

#### Electric stimulation setup

Stimulation waveforms (sinewave) were generated in a custom written Matlab (Mathworks, Natwick, MA) 2014a based software. The waveforms were sent to a data acquisition card (NI USB-6216, National Instruments, Austin, TX) which then sent the signal as voltages to an AM 2200 analog current source (AM Systems, Sequim, WA). The negative and positive terminals of the current source stimulator were attached to the center disc of the electrode closest to the craniotomy and the outer ring of the same electrode for focused stimulation. Focused stimulation always targeted the craniotomy hemisphere and allowed us to explore targeted stimulation. For unfocused stimulation the two outputs were attached to the center discs of the electrodes on both sides of the head and allowed us to chart out neural responses across the brain when stimulation is not targeted.

#### Recording setup

On experiment days a standard recording grid (Crist Instruments) was fixed inside the recording chamber. The entry positions of the recording grid gave a recording trajectory in the brain. A single tungsten electrode (FHC, impedance as reported by manufacturer: ~1 MΩ at 1 kHz) was then advanced (FHC hydraulic microdrive) into the brain within a stainless-steel guide tube.

Recordings were amplified (100x) through a regular BAK Electronics preamplifier (Model A-1). The amplified and unfiltered signals were recorded via the previously mentioned data acquisition card at a sampling rate of 20 kHz and stored for offline analysis. Recordings were visualized online using the earlier mentioned Matlab based software.

#### Experimental protocol

After insertion we advanced the electrode into the cortex until we reached a site with clear and robust multi-unit spiking activity. We refer to these positions as “recording sites”. We then started the stimulation experiment which consisted of a trial of two consecutive minutes of neural activity recordings during passive fixation. No stimulation was delivered during the first minute, during the second minute we stimulated (without ramping) through the concentric ring electrodes at 10 and 40 Hz﻿ (see Figures 1B,F,J). We stimulated at 0.25, 0.5, 1, 2 and 3 mA for the unfocused and 0.25, 0.5, 1, 2, 3 and 4 mA for the focused. After these two minutes the trial was terminated. Approximately 10 s thereafter we started a new trial at the same recording site with different parameters. The choice for a frequency-amplitude combination was pseudo-randomized so that each combination occurred only once. We then advanced the recording electrode deeper by at least 500 μm to find a new recording site. Across all experimental sessions we recorded from brain depths ranging from 58 to 10,150 μm.

#### Electro-anatomical model and electric field calculation

To determine generated E-fields we first used an electro-anatomical computational head model of the monkeys to estimate electric field distributions. We then validated the computational model using experimentally acquired electric potential measurements obtained in monkey P.

#### Computational electric field estimation

We created a computational head model for each of the two monkeys to estimate the electric field distribution during stimulation. For each monkey, a pre-operative MRI scan (slice thickness: 0.6 mm, Siemens 3 T scanner) and a post-operative CT scan (0.3 mm resolution) were acquired. The MRI was used to segment the brain and the CT was used to segment skull and skin. For segmentation the MRI was imported into Freesurfer (7.1.0) where the brain was first extracted using the “bet” function. White matter (WM) was then segmented using the “mri_segment” function and finally the function “recon-all -autorecon2-wm” was called to segment grey matter (GM). To separate the skin and the skull the CT was imported into ScanIP and intensity thresholding applied.

The MRI scan and the segmented masks were then imported into ScanIP 7 (Simpleware Ltd., Exeter, UK) and registered to the CT using anatomical landmarks. The skull and dental cement were separated ScanIP’s “magnetic lasso.” The CSF was segmented by filling the area between the skull and GM. The metal stimulation electrodes caused an artefact in the image which showed up as high value regions. To model our stimulation electrodes we applied a high intensity threshold in the area where the electrodes had been implanted. This procedure effectively isolated a rough model of the stimulation electrodes. We then optimized and smoothed the model of the stimulation electrodes by fitting a cylinder to the isolated model. Using ScanIP volumetric tetrahedral meshes were generated which were then imported into COMSOL multiphysics 5.3 (COMSOL, Inc., Burlington, MA). The electric conductivities of the different model parts were set as follows: CSF (1.65 S/m), GM (0.27 S/m); cerebellum (0.2 S/m); WM (0.127 S/m); Skull (0.01 S/m); dental cement (0.01 S/m); electrode contacts (5*10^7^) (Akhtari et al., 2002; Datta et al., 2009; Gabriel et al., 2009; Peters et al., 2001). Finally, the distributions of the electric potential (*φ*), fields (*E*) as well as densities (*J*) were estimated for each 1 mA of stimulation by defining boundary conditions and solving Laplace’s ﻿(Equations 1–3).


(1) ∇⋅σ∇φ=0

(2) E=∣∇φ∣

(3) J=σ∣E∣

This assumes a quasi-static approximation of Maxwell’s equations; a valid approach for electric fields in the brain with frequencies below 1 MHz (Nunez and Srinivasan, 2006).

### Electric field validation

#### Experimental extraction of electric potentials

For monkey P, at the end of each recording day while retracting the electrode from the brain we stopped every 500 μm (these are ‘E-field recording depths’) and stimulated at 10 Hz and at multiple stimulation amplitudes for 3 s while recording. We stimulated through the focused as well as the unfocused montages, separately. This procedure was repeated until the electrode was out of the brain. During the electric stimulation the recorded neural signal took on the shape of the sinewave stimulus ﻿(we refer to this as stimulation-artifact, see Figures 1C,G,K). electric stimulation caused a distortion of the recorded signal which we used for the E-field calculations. We used this stimulation artifact to calculate E-fields by quantifying the amplitude of the stimulation-artifact in mV at the different electric field recording depths. For the validation the amplitude of the stimulation-artifact was always adjusted to a stimulation amplitude of 1 mA; by dividing the stimulation-artifact amplitude over the stimulation amplitude. Thus, we obtained experimental electric potential values along the path of every recording grid trajectory. We could then compare these recorded electric potential values to those extracted from an identical trajectory in the electro-anatomical model. Comparison of the recorded and modeled electric potential values then served as a method to validate the accuracy of the electro-anatomical model.

During the post ‘second surgery’ MRI and CT scan we filled a glass capillary with a 2% copper sulfate solution and inserted it in the center position of the recording grid. In the CT images the capillary appeared as white and had the highest intensity values. We isolated the position of the capillary by extracting these high intensity areas which gave a trajectory through the brain. This center trajectory served as reference for all recording trajectories.

#### Electric field value assignment to neural recordings

To assign an electric field value to a neural recording we took the recording trajectory as well as the recording depth of a particular recording and extracted at this point in the electro anatomical head-model the E-field value for that recording. This electric field value was multiplied by the stimulation amplitude during the experimental recording. We then assigned this value to that experimental recording.

### Data analysis

#### Spike times extraction

We filtered recorded signals (300–3,000 Hz) with a second order Butterworth filter. This also removed the stimulation-artifact (10 or 40 Hz) so that we could asses stimulation effects on spiking. We also discarded on- and offset noise (Figures 1D,H,L; Supplementary Figure 1). In a three-step cross-correlation approach (Franke et al., 2015; Kim and McNames, 2007; Laboy-Juárez et al., 2019) we extracted spiketimes by generating an average spike, then cross correlating this with the filtered signal and lastly extracted spiketimes. In detail spike time extraction was as follows: (1) average spike generation: We applied amplitude thresholding by estimating the standard deviation of background noise with Quiroga et al. (2004)﻿ (Equation 4).


(4) $$\sigma_{e}=\text{median}\frac{|x|}{0.6745}$$

Where *x* is the filtered signal and the denominator is derived from the inverse of the cumulative distribution function of the standard normal distribution evaluated at 0.75 (Donoho and Johnstone, 1994). To determine spikethreshold we multiplied *σ_e_* by −4 and averaged all signals that passed this threshold to get an intermediate average spike. We then calculated the sum of squares differences (SSD) of every detected signal in relation to the intermediate average spike. All signals whose SSD were below 2.5 were considered spikes and were then averaged to get the average spike for the next step of spike extraction. (2) cross correlation: We cross-correlated this average spike with the filtered signal. (3) Spiketime extraction: We thresholded the obtained cross-correlation signal (3*σ_e_) and recorded timestamps where the cross correlation signal passed the threshold. To reject possible artefacts timestamps whose peak value exceeded 3 z-scores of the cross correlation signal were rejected. This procedure yielded multi-unit spiking activity which we applied further analysis to.

#### Entrainment calculation

Firstly, we separated extracted spiketimes in a stimulation-OFF and ON conditions. For stimulation-ON we binned spikes (according to the phase where they occurred) into 30 phase-bins and normalized (by dividing the number of spikes per bin over the total number of spikes) across the condition to get rates per bin. For stimulation-OFF we assumed the period of the stimulation-ON condition and repeated the procedure. This means we virtually extended the stimulation waveform to cover the time period before stimulation onset. We then calculated phase lock value ﻿(PLV, adjustment of spiketimes to stimulation phase, Equation 5)


(5) $$\mathrm{PLV}=|\;\sum_{b}R_{b}e^{i\theta_{b}}\;|$$

where θ*_b_* is the center of bin ‘b’ and denotes the phase of the cycle; *R_b_* is the magnitude of bin ‘b’. For PLV, *R_b_* was the probability that a spike occurred in bin ‘b’. The PLV metric runs from 0 to 1 where 0 means there is no entrainment (spikes were equally likely to fall in all bins) and 1 means there is absolute entrainment (i.e., all spikes fell into one bin). To get a measure of entrainment we subtracted the PLV of the stimulation-OFF condition from the PLV of the stimulation-ON condition﻿ (see Figures 1E,I,M).

#### Statistics

We employed the Wilcoxon signed rank to compare groups. To track neural response over stimulation intensities and generated E-fields we applied linear mixed models with subject as random and stimulation intensity or electric field as fixed effects. Multiple comparisons were corrected using the Bonferroni approach. *α* was set at 0.05. In the results section we further clarified which precise comparisons were done and provide the mixed model structure. Full statistics are shown in the figures they belong to.

### Functional magnetic resonance imaging (fMRI) experiments

#### Preparation

For the fMRI experiments, carbon fiber rubber electrodes (Neurocare, Munich, Germany) were implanted at the same position as the concentric ring electrodes in monkey D. These electrodes minimized artefacts thereby allowing visualization of tissue immediately under the electrode. In monkey P., we used the concentric ring electrodes from the electrophysiological recordings. For this fMRI study we only used the unfocused montage. On scanning days monkeys were sedated with a mixture of ketamine (Nimatek, Eurovet, 12.5 mg/30 min) and medetomidine HCL (Domitor, Orion, 0.25 mg/ 30 min) in the ratio 2:1. A contrast agent monocrystalline iron oxide nanoparticle (Faraheme AMAG pharmaceuticals, 11 mg/kg) was then injected into the femoral/saphenous vein (Vanduffel et al., 2001).

#### Image acquisition, electric stimulation and protocol

Monkeys were placed in a 3.0 T full body scanner (PrismaFit, Siemens; Erlangen, Germany). We used a gradient-echo T2* weighted EPI sequence (40 horizontal slices, TR 2 s, TE 16 ms, 1.25mm^3^ isotropic voxels) with a custom built 8-channel phased-array receive coil and a saddle shaped radial transmit-only surface coil (Ekstrom et al., 2008). While in the scanner the implanted stimulation electrodes were attached to the aforementioned current source. The stimulation setup was identical to earlier described. During data acquisition periods of 30 s stimulation-ON and 40 s stimulation-OFF were alternated. We stimulated, always using the unfocused montage (since this montage increased spike rate), with a sinewave of 10 Hz and amplitudes of 1, 2 and 3 mA for each acquisition day separately.

#### Data analysis

The EM-fMRI experiments were analyzed using statistical parametric mapping (SPM12) using a fixed-effects GLM. Spatial preprocessing consisted of rigid co-registration with the animal’s own anatomical scan. The functional volumes were then resliced to 1 mm^3^ isotropic and smoothed with an isotropic Gaussian kernel (full width at half maximum: 1.5 mm). Single subject analyses were performed, and the level of significance set at *p* < 0.001, uncorrected for multiple comparisons as in previous studies (Premereur et al., 2015).

## Results

**Figure 2:**
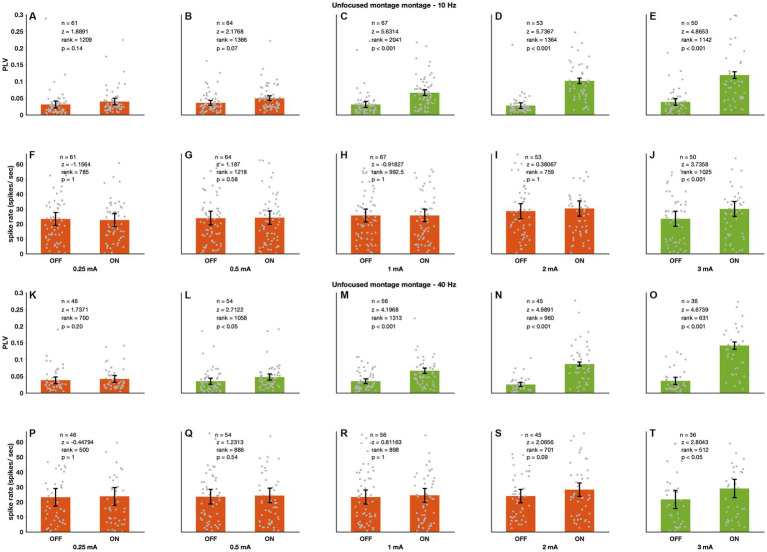
Unfocused sinewave stimulation causes entrainment and spike rate changes. During experiments we recorded spiking activity 1 min without stimulation (OFF condition) followed by another minute with a sinewave stimulation (ON condition) and compared the two. **(A–E)** 10 Hz stimulation caused entrainment from 1 mA upwards. **(F–J)** The stimulation also increased spike rate but only at the highest stimulation amplitude tested (3 mA). 40 Hz stimulation (**K–O**: entrainment; **P–T**: spike rate) showed the same trend. However, entrainment started at an earlier stimulation amplitude (0.5 mA). Monkey D showed lower response thresholds (see discussion section “Effects of montage and frequency”). There are 90 recording sites for the 10 Hz and 73 recording sites for the 40 Hz stimulation. Green bars depict statistically significant stimulation parameters and orange bars the non-significant parameters.

**Figure 3:**
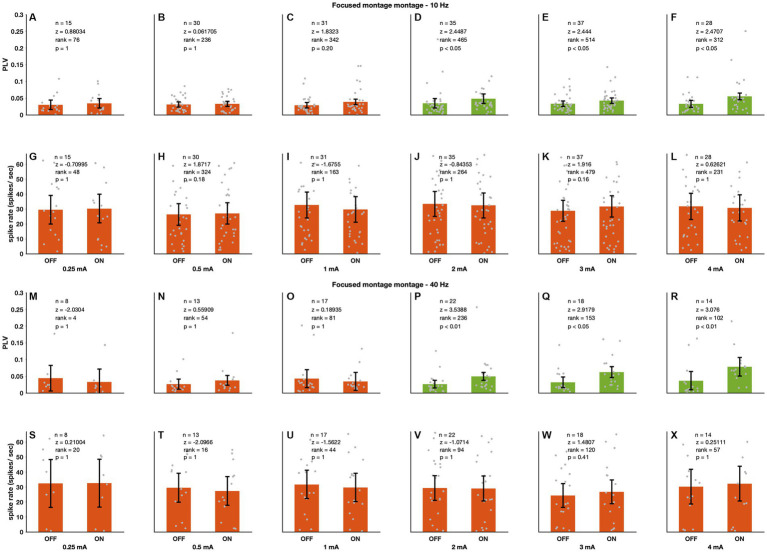
Focused sinewave stimulation causes entrainment. This figure follows the same convention as Figure 2. **(A–F, M–R)** 10 and 40 Hz stimulation respectively caused entrainment from 2 mA on. **(G–L, S–X)** In contrast to unfocused stimulation however this montage did not cause spike-rate changes for 10 (row 2) nor 40 (row 4) Hz stimulation. There are 47 recording sites for the 10 Hz and 25 recording sites for the 40 Hz stimulation.

To investigate the neuromodulatory effect of epicranial stimulation (ECS) we recorded spiking activity in the parietal convexity while stimulating via concentric ring electrodes implanted on the skull of two rhesus monkeys above PFG in both hemispheres. This montage allowed focused (*n* = 72 recording sites) as well as unfocused (*n* = 163 recording sites) stimulation. We stimulated with sinewaves of 10 (*n* = 90 sites unfocused and 47 sites focused) and 40 Hz (*n* = 73 sites unfocused and 25 sites focused) and amplitudes ranging from 0.25 to 4 mA. We recorded from a total of 105 recording sites (55 in monkey P and 50 in monkey D). For the number of recording sites per frequency-amplitude combination, see Figures 2, 3. In this study we report recording sites since the single recording electrode only allowed the analysis of multi-units. The number of reported recording sites are also the number of samples.

### Effects of unfocused ECS on PFG neural spiking

Figure 2, shows group results of unfocused 10 and 40 Hz stimulation from 0.25 to 3 mA (10 Hz *n* = 90, 40 Hz *n* = 73); higher stimulation intensities tended to increase entrainment and spike rates (PLV – 10 Hz – 1 mA: *p* < 0.001, 2 mA: *p* < 0.001, 3 mA: *p* < 0.001; 40 Hz – 0.5 mA: *p* < 0.05, 1 mA: *p* < 0.001, 2 mA: *p* < 0.001, 3 mA: *p* < 0.001; spike rate – 10 Hz – 3 mA: *p* < 0.001; 40 Hz – 3 mA: *p* < 0.05; Wilcoxon sign rank; see figure for full statistics). 10 and 40 Hz entrainment were similar across stimulation intensities (Supplementary Figures 3A,B; Wilcoxon sign rank, see Figure for full statistics). A linear mixed model [*PLV ~ stimIntensity + (1|subject)*] showed that entrainment increased with increasing stimulation intensity (entrainment-10 Hz z = 54.49 *p* < 0.001, 40 Hz z = 19.97 *p* < 0.001) (Supplementary Figures 2A,B).

We calculated spike rate changes (*Δ* spike rate) by subtracting the stimulation-OFF rates from the stimulation-ON rates. For both 10 and 40 Hz higher intensities tended to cause spike rate increases. Furthermore, a linear mixed model [*spikeRate ~ stimIntensity + (1|subject)*] analysis showed that spike rate increase depended, for both 10 and 40 Hz, on stimulation amplitude (spike-rate-10 Hz z = 54.49 *p* < 0.001, 40 Hz z = 19.97 *p* < 0.001) (Supplementary Figures 2C,D).

### Effects of focused ECS on PFG neural spiking

Figure 3 shows the effects of focused stimulation at amplitudes from 0.25 to 4 mA (2 upper panels 10 Hz *n* = 47 and 2 lower panels 40 Hz *n* = 25). At 2 mA neural activity became entrained to the stimulation (10 Hz – 2 mA: *p* < 0.05, 3 mA: *p* < 0.05, 4 mA: *p* < 0.05; 40 Hz – 2 mA: *p* < 0.01, 3 mA: *p* < 0.05, 4 mA: *p* < 0.01; Figures 3A–F for 10 Hz and M-R for 40 Hz). However, in contrast to unfocused stimulation this montage did not affect spike rate, even at 4 mA (Wilcoxon sign rank, see Figures 3G–L,S–X for full statistics). Furthermore, 40 Hz focused stimulation caused comparable levels of entrainment to the focused 10 Hz (Supplementary Figure 3B, Figure contains full statistics).

**Figure 4:**
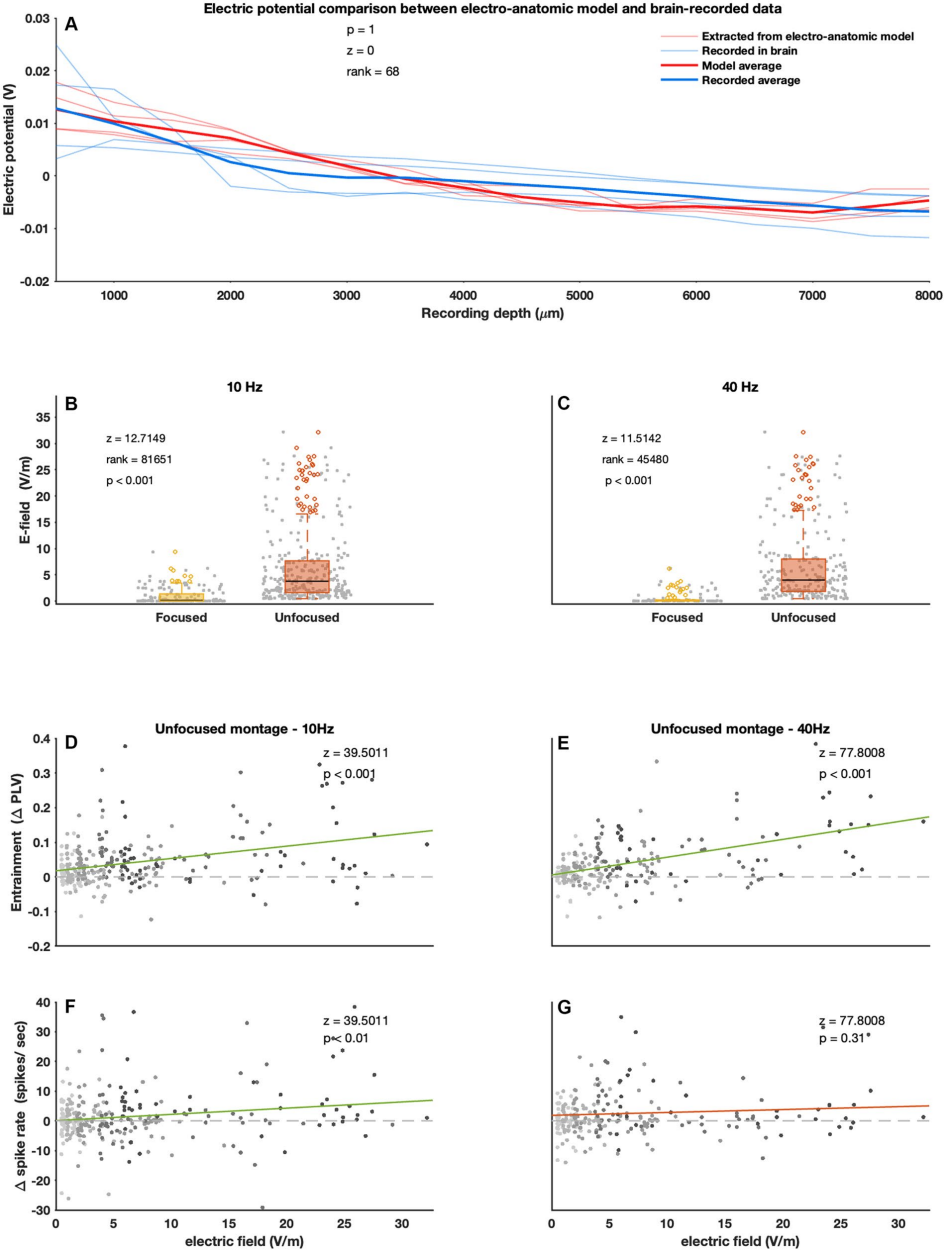
Electric field generated by ECS and its relationship to entrainment and spike rate. **(A)** Experimentally acquired electric potentials and computationally calculated potentials were comparable. The graph shows electric potential as a function of recording depth. The light red lines are calculated electric potentials of different recording trajectories. The different trajectories were averaged on the basis of recording depth to get the bold red line. The light blue lines are experimentally acquired values (from the same trajectories as the light red lines) and was similarly averaged (bold blue line). The averaged values were then compared using the Wilcoxon signed rank test. **(B)** Unfocused (orange) ECS generates stronger electric field values compared to focused (yellow) ECS. During unfocused stimulation electric field values tended to be higher than during focused stimulation. Both 10 **(B)** and 40 **(C)** Hz showed this effect. **(D,E)** Entrainment levels are shown as a function of electric field values. For both 10 **(D)** and 40 **(E)** Hz stimulation entrainment levels increased as electric field strength increased. **(F,G)** The y-axis shows *Δ* spike rate; here 10 Hz stimulation also showed a significant slope (although this is a small effect) while the 40 Hz did not. E-field values are adjusted to a stimulation intensity of 1 mA (see section “experimental extraction of electric potentials”). Every dot represents a metric (**B,C**: E-field, **D,E**: entrainment, **F,G**: Δ spike rate) at one recording site.

At amplitudes where entrainment was observed in both montages (i.e., 2 and 3 mA) the level of neural entrainment was higher in the unfocused montage (Supplementary Figure 3C, 3 at 2 mA *p* < 0.001, at 3 mA, *p* < 0.001, panel 4 at 2 mA *p* < 0.001, at 4 mA *p* < 0.001 see figure for full statistics; Wilcoxon sign rank). This is likely due to the stronger electric field values in the brain with the unfocused montage (Figures 4B,C at 10 Hz *p* < 0.001, at 40 Hz *p* < 0.001, Wilcoxon sign rank; see Figure for full statistics).

### Effects of generated electric field on entrainment and spike rate

Figure 4A shows good agreement between model estimated values electric potential values and the measured electric potential in monkey P. Figures 4B,C shows the model estimated electric fields at the unfocused recording sites was significantly larger than at the focused sites (Wilcoxon sign rank 10 Hz *p* < 0.001, 40 Hz *p* < 0.001). A linear mixed model analysis showed that entrainment increases with increasing electric field strength for the unfocused montage at both 10 [*PLV ~ eField + (1|subject); p < 0.001*] and 40 [*PLV ~ eField + (1|subject); p < 0.001*] Hz (Figures 4D,E). A similar effect was found for spike rate [*spikeRate ~ eField + (1|subject)*] but only with 10 Hz (*p* < 0.01) stimulation (Figures 4F,G). Overall, focused stimulation did not show this electric field dependency on entrainment and spike rate effects. Spike rate at 40 Hz did increase with increasing electric field strength (*p* < 0.001), although this might be driven by an outlier (see Supplementary Figure 4 for all statistics).

### Functional activations during ECS

**Figure 5:**
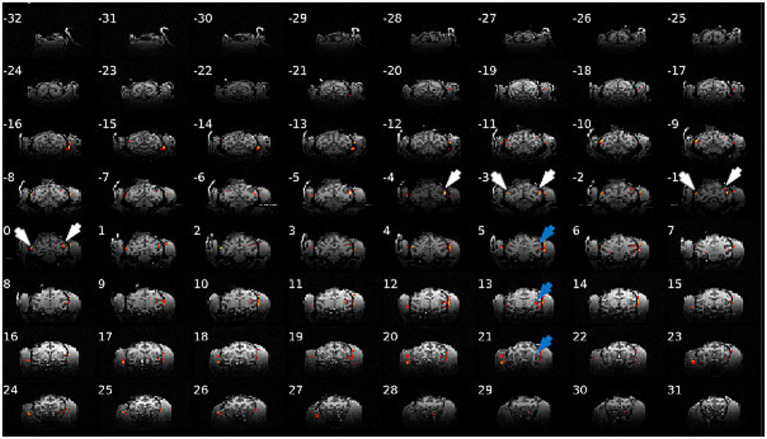
Brain activation in monkey D during ECS sinewave stimulation (10 Hz, 3 mA). ECS can activate target areas under the electrodes (white arrows) in addition to a series of other cortical sites (blue arrows).

Figure 5 shows the fMRI activations caused by unfocused ECS stimulation at 10 Hz and 3 mA in monkey D. ECS activated a relatively restricted region under the stimulation electrodes (white arrows in Figure 5), but also in the Lateral and Superior Temporal Sulcus (STS, blue arrows). Note that the activations were located slightly lower and more anterior in the left hemisphere, consistent with the lower and more anterior position of the stimulation electrode on that side. Supplementary Figure 5 shows a similar figure for monkey P. However, except in one session, ECS at 1 and 2 mA did not evoke activations (data not shown). These results demonstrate that ECS at 3 mA can activate the target area under the electrodes in addition to a series of other cortical sites. Furthermore, using fMRI it is possible to visualize this activity.

## Discussion

ECS is a novel neuromodulation method in which an electrode is implanted under the scalp on the skull. It is more invasive than TES, but less invasive than DBS or ICS as no craniotomy nor opening of the dura is required. Combined with techniques as Intersectional short duration pulses (ISP) or interfering fields (IFS; which, in line with this work, uses sinewaves) these properties make ECS a potentially useful neuromodulation therapy for a wide range of disorders, e.g., Parkinson’s disease, depression, obsessive-compulsive disorder, epilepsy, and chronic pain. In this study we investigated the neural effects of ECS in parietal cortex of awake rhesus monkeys. ECS induced robust entrainment of neural populations at low stimulation amplitudes without affecting spike rate. At higher stimulation amplitudes ECS caused higher levels of neural entrainment which were accompanied with increases in spike rate (Figures 2, 3). In line with the computational model predictions, the electric fields generated in the brain during ECS were an order of magnitude higher than can be expected with TES at an equivalent current amplitude [Supplementary Figure 2; see Khatoun et al. (2018a, 2018b) and Vöröslakos et al. (2018)]. Our results showed that an unfocused ECS montage caused entrainment at relatively low current amplitudes (from 0.5 mA), while higher current amplitudes (from 2 mA) were needed to cause entrainment with the focused ECS montage (Figures 2, 3). Moreover, ECS during fMRI revealed a distributed pattern of activations throughout the brain during ECS at higher stimulation intensities (Figure 5; Supplementary Figure 5).

### ECS electric field strength

This study provides the first in-vivo evidence that ECS generates strong E-field ranging from 1 to 30 V/m (depending on distance to stimulation-electrode) in the macaque brain (Figures 5D–G). We expect lower values in humans due to the thicker skull. On the other hand, DBS (Huang et al., 2017; Lafon et al., 2017) and TES (Khatoun et al., 2019b; McIntyre et al., 2004) where, respectively, 100 V/m and 0.15 V/m are generated at the same 1–2 mA intensity.

Neural response to ECS was linear with higher stimulation intensities causing larger responses (Supplementary Figure 2). This revealed a wide range of neuromodulation from small entrainment [similar to TES (Krause et al., 2019; Ozen et al., 2010)] levels at low stimulation intensities to robust spike rate increase [similar to DBS (McIntyre et al., 2004)] with high intensities (Figures 2, 3).

The linear neural response is in line with many electric modulation paradigms (Asamoah et al., 2019; Vieira et al., 2020; Vöröslakos et al., 2018). Although we expect that further increase would truncate this linearity; however this was not tested. It should be noted that some electric neuromodulation studies (mostly using direct current) have reported (partially) non-linear effects (Batsikadze et al., 2013; Vimolratana et al., 2023). It remains unclear how the non-linearity relates to this study given the particular conditions of this work.

### Effects of montage and frequency

Observed effects between focused and unfocused montage were mostly similar although there were some differences. Most notably the unfocused montage induced stronger E-fields and spike rate as well as entrainment responses depended on stimulation amplitude and E-field strength (Figures 4D–G; Supplementary Figure 2). This relationship was not as clear for the focused montage (Supplementary Figure 4) which tends to generate weaker and more focused E-fields in the brain (Khatoun et al., 2018a, 2018b). Recording positions may sometimes have been sub-optimal in relation to the stimulation electrode which targeted a small brain area. It is thus possible that the focused montage also causes amplitude dependent effects in the stimulation intensity range we investigated.

40 Hz unfocused stimulation appeared to cause entrainment at lower stimulation intensities (in Figures 2B,L entrainment starts at 0.5 mA for 40 Hz and 1 mA for 10 Hz). A direct comparison however showed this frequency difference was not significant (Supplementary Figures 3A,B). Notably, Monkey D seems to have a lower response threshold as compared to Monkey P (see stimulation amplitude 0.5 mA in Supplementary Figures 6, 7 and Figure 2). This is in line with other non-invasive neuromodulation where differences between individuals have been reported (Hsu et al., 2016; Santarnecchi et al., 2016; Zanto et al., 2021). It would be interesting to test whether other brain regions would respond differently. For example, in the hippocampus where gamma band plays a central role in memory encoding (Lisman and Jensen, 2013) 40 and 10 Hz stimulations may elicit differential responses.

### ECS-fMRI

A crucial advantage of our ECS approach in monkeys is that we could chart the effects of ECS throughout the brain by means of an fMRI study, where we only used the unfocused montage. Numerous previous studies using intracortical stimulation during fMRI have furnished invaluable insights into the organization of cortical networks underlying face (Moeller et al., 2008), body (Premereur et al., 2016), attention (Ekstrom et al., 2008), and 3D shape processing (Van Dromme et al., 2016). We observed localized activations in the cortex immediately below the stimulation electrodes rather than the diffuse activations that may have been expected from the modeling [Figure 1A (inset)]. This observation is highly similar to the results of a previous TMS study in monkeys (Romero et al., 2019), which measured highly localized spiking responses in a 2 by 2 by 2 mm volume of cortex under the TMS coil despite the widespread electric field effects predicted by modeling. A thresholding phenomenon may explain why the activations were localized under the stimulation electrode (note that we also did not observe any fMRI activations at 1 or 2 mA.)

The fMRI study showed that ECS activates areas directly under the electrodes as well as at remote locations (Figure 5; Supplementary Figure 5). Interestingly these remote activations were localized implying they were not immediately caused by the generated electric field in the brain or conduction through the cerebrospinal fluid. Rather, they were activated indirectly by the areas that were directly activated by the stimulation. Future studies investigating the behavioral effects of ECS will also benefit from ECS-fMRI for the interpretation of results.

### Opportunities

DBS and ICS are effective therapies; however, due to their high cost and invasive nature, they are only considered in advanced disease stages (Kennedy et al., 2011; Krack et al., 2019; Lozano et al., 2019; Tsubokawa et al., 1993). These considerations are minimized in the portable ECS system (﻿Kravalis and Schulze-Bonhage, 2020; Schulze-Bonhage et al., 2023) where electrodes are implanted in the subgaleal space between the skin and the skull. Through technological advancement ECS implantation will likely require only an incision in the scalp under local anesthesia thereby siginificantly reducing cost, risk and discomfort to the patient. As a result ECS could be considered at a much earlier disease stage than more invasive neuromodulation methods. In a current clinical trial epilepsy patients receive subthreshold stimulation with the option to self-administration; current results show a promising development (Schulze-Bonhage et al., 2023).

On the other hand, TES has been investigated as a noninvasive treatment for a range of neurological and psychiatric disorders (Mehta et al., 2015; Palm et al., 2016). Its advantage is that it can be applied at a much earlier disease stage. However, it is not yet an accepted therapy, possibly because of reproducibility issues caused, among others, by weak E-fields in the brain (Asamoah et al., 2019; Lafon et al., 2017). In a subpopulation of these patients ECS can be applied more effectivity. More studies (animal models and clinical trials) are needed to fully explore the potential of ECS as a neuromodulation therapy. Intersectional short duration pulses (ISP) or interfering fields (IFS) are novel neuromodulation approaches which may bypass neurons in superficial layers (Grossman et al., 2017; Vöröslakos et al., 2018). In a recent computational study (Khatoun et al., 2021) we showed that these novel approaches combined with ECS allow for stronger and more focused E-fields in subcortical regions than TES.

Our ECS approach combined with extracellular recordings showed that moderate stimulation intensities (1–2 mA) induce robust neural entrainment without changing the spike rates of the neurons. This observation opens the possibility to study the neural and behavioral effects of pure neural entrainment at different frequencies in a cortical area under the stimulation electrode. Moreover, this robust entrainment means that applying in-phase or out of phase ECS in two distant but interconnected areas may provide a critical test of the communication by coherence hypothesis (Fries, 2015), which states that interareal communication is improved if two areas oscillate together. With further carefully designed clinical trials we can better understand how to use the different montages to target very specific brain region (e.g., in DBS) or induce a more network response as the fMRI experiment showed.

### Limitations

A limitation of ECS is that it requires surgery, possibly under local anesthesia. As such, it could not be used to probe neural mechanisms in healthy volunteers. Furthermore, our ealier work shows that insulation can prevent current flow between implanted electrodes and prevent stimulation of scalp and cervical nerves. Nevertheless, it can still stimulate dura nerves (Lv et al., 2014). By choosing the right stimulation intensity or approach (i.e., ISP and IFS) this co-stimulation can be mitigated while achieving strong E-fields in the brain.

In this study we implanted the electrodes on the monkey’s skulls and conducted experiments during a period of approximately nine months per monkey. In this period the electrodes functioned and caused no obvious damage to the skull or neural tissue. For ECS to function as a long duration neuromodulatory approach, stimulation electrodes will be implanted for prolonged periods of time (years). This work however does not provide direct insight into this aspect of ECS. However, deep brain stimulation has been applied as a therapy for decades (Delgado et al., 1952; Sironi, 2011), it is well tolerated, reversible and unknown to cause brain damage (Krack et al., 2019; Nuttin et al., 1999). Previous rodent studies have charted damage as a response to stimulation (Grossman et al., 2017). Future animal studies as well as ongoing clinical trials may give deeper insights into tissue responses to prolonged stimulation of skull and brain.

We note that for the fMRI section of this study the monkeys were anesthetized. Anesthesia generally may induce inhibition and reduced excitation of neural activities (Kitamura et al., 2003). Activity may show increased correlation in functional areas while activity between functional areas may be decreased (Bettinardi et al., 2015; Ordek et al., 2012). We therefore expect the fMRI results to differ from awake responses. Notably, neural activity recorded under anesthesia remains active and informative. Further studies can determine the extent to which awake responses may be different from anesthetized responses in ECS.

## Conclusion

In this study we investigated for the first time the neuromodulatory effects of the novel ECS. We showed that ECS entrains neurons (similar to TES) and causes spiking (similar to DBS) when stimulation amplitude was low or high, respectively. We investigated its diversity by using a focused – to target a spatially restricted region – and an unfocused – to target broader regions – montage. The unexpectedly distributed pattern of activations, revealed by fMRI, implies ECS may have activated network responses. Further studies are needed to understand the implication of these neural responses for neuromodulation and therapy applications.

## Data Availability

The raw data supporting the conclusions of this article will be made available by the authors, without undue reservation.
