# Supplementary material for: Neuronal effects of epicranial current stimulation in macaque cortex
Source: Front Neurosci. 2025 Sep 23;19:1627705. doi: 10.3389/fnins.2025.1627705 (PMC12500610; doi:10.3389/fnins.2025.1627705)
Supplement: Supplementary file 1 [file Data_Sheet_1.PDF]

Supplementary document

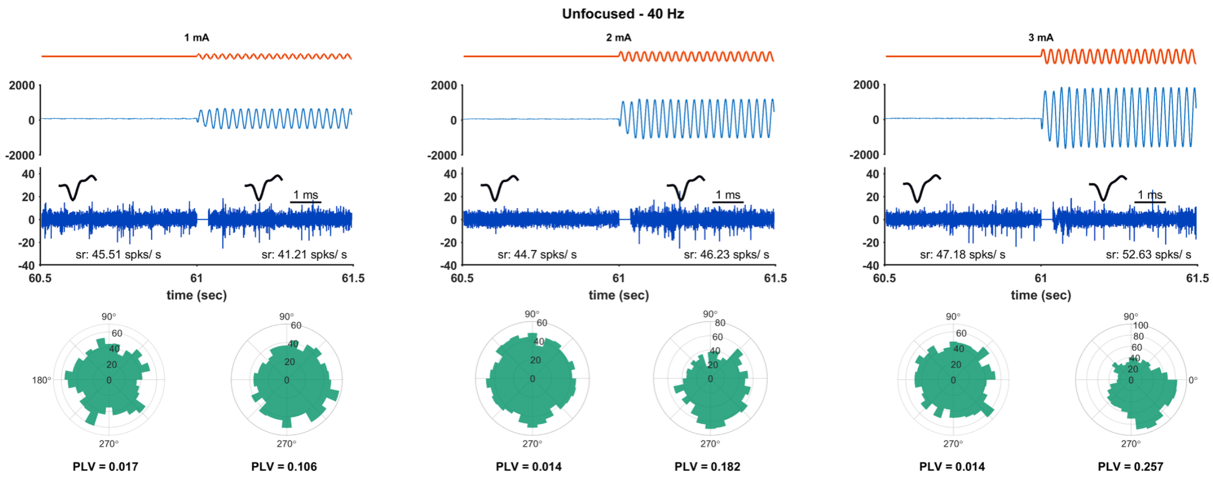

Supplementary Figure 1: Response of an example recording site (same as in Figure 2 in main text) to 40 Hz sinewave stimulation at increasing stimulation amplitudes. Same convention as in Figure 2. Notice that this same recording site entrains to a different angle (as compared to Figure 2) and spike rates appear to be unchanged.

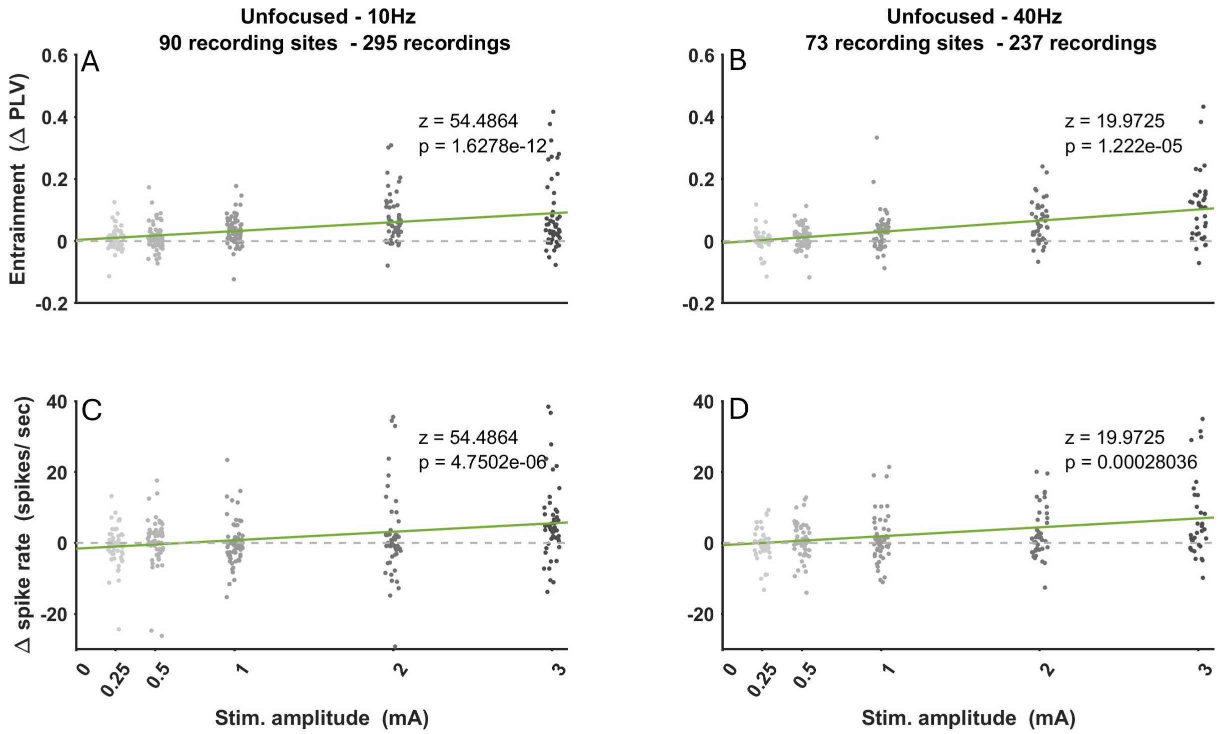

Supplementary Figure 2: Entrainment and spike rate increase with increasing stimulation amplitude. In the ***upper row*** *entrainment levels are shown as a function of stimulation amplitude. For both 10 (upper left) and 40 (upper right) Hz stimulation as stimulation amplitude was increased entrainment levels also became larger. This trend was also seen for the spike rate effect in the* ***lower row*** *where the y-axis shows Δ spike rate as a function of stimulation amplitude. The number of neurons per amplitude group as well as number of unique neurons is the same as in Figure 3.*

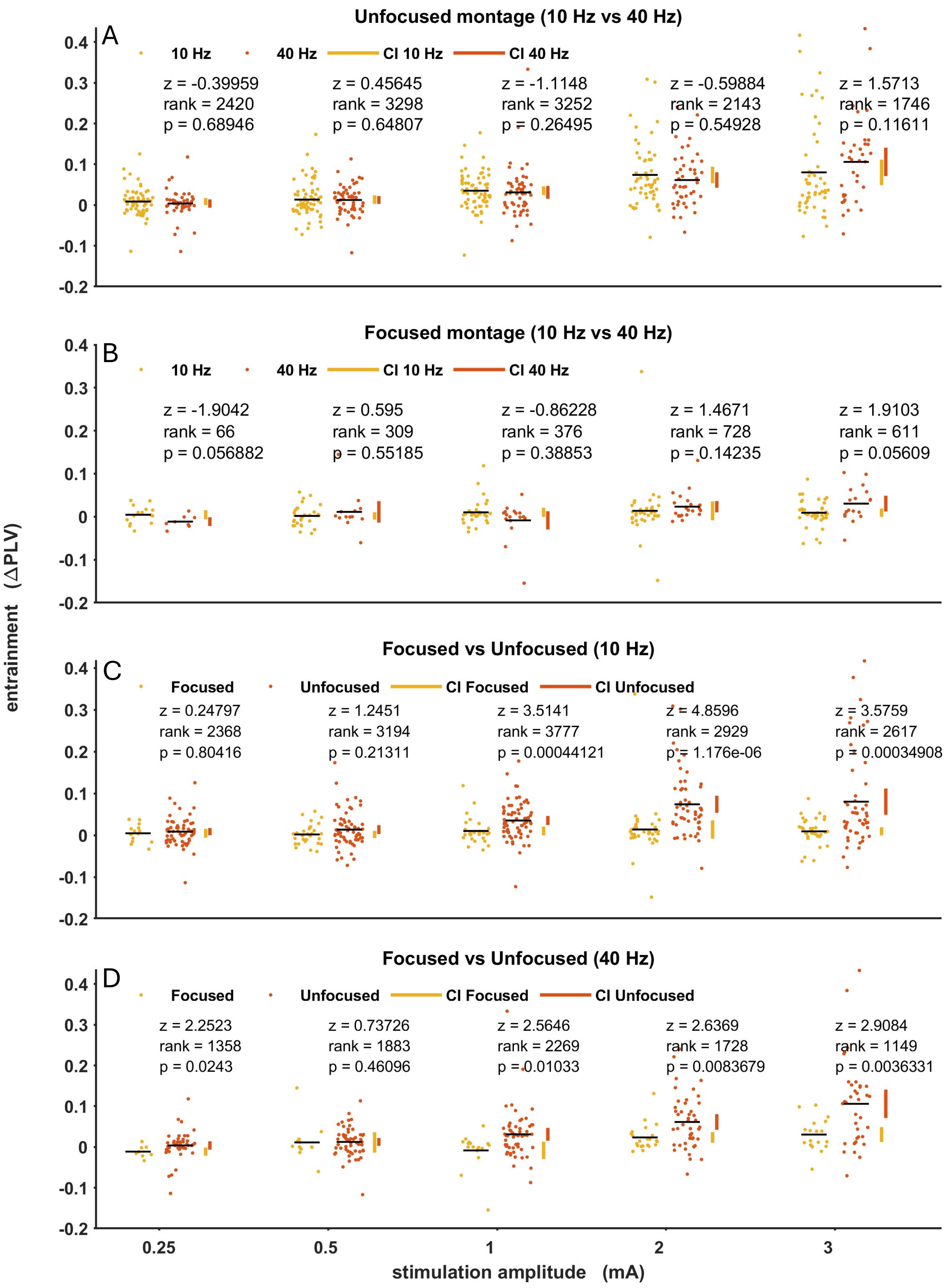

Supplementary Figure 3: Comparison of entrainment levels on the basis of electrode montage and stimulation frequency using Wilcoxon sign rank. Every datapoint is entrainment as a function of stimulation amplitude. Yellow represents 10 and orange 40 Hz stimulation. The horizontal black bars show the mean and the vertical bars show

*95% confidence intervals. Entrainment values caused by 10 and 40 Hz stimulation are compared for the unfocused montage in **panel 1** and the focused montage in **panel 2**. Entrainment caused by the two frequencies were comparable. Entrainment caused by the focused and unfocused montages are compared in **panel 3** for 10 Hz stimulation and **panel 4** for 40 Hz stimulation. From stimulation amplitudes of 1 mA and higher the unfocused montage caused higher entrainment levels as compared to the focused montage.*

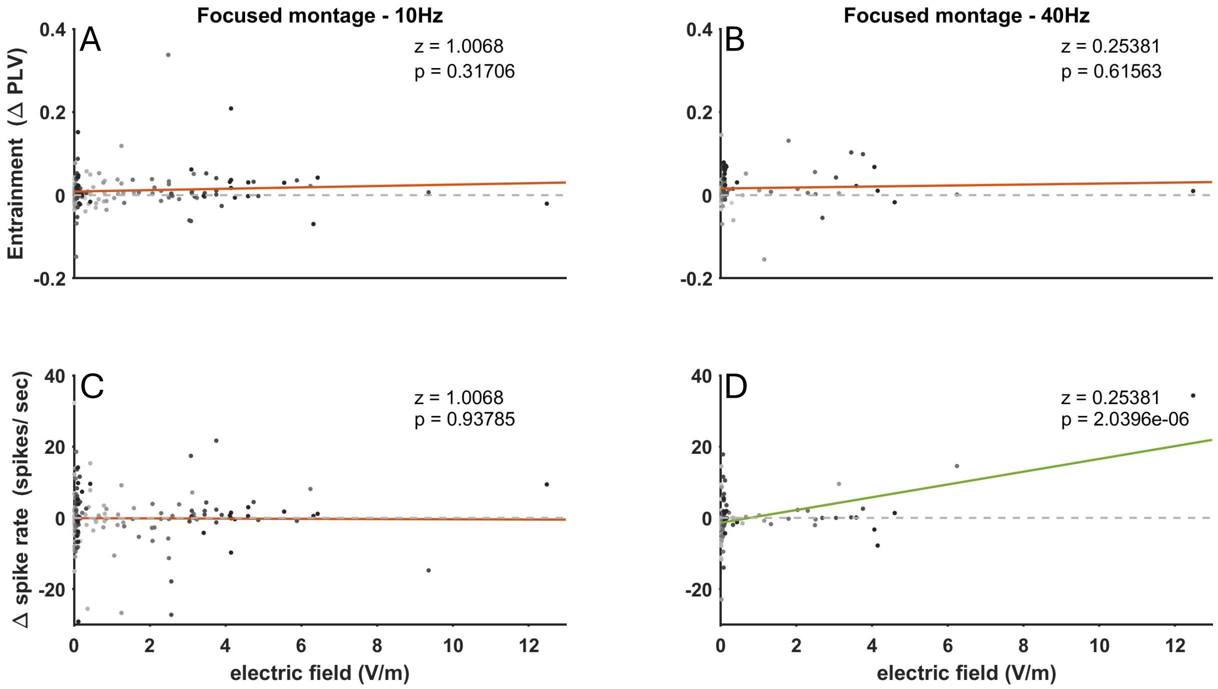

*Supplementary Figure 4: Overall stimulation effects did not depend on electric field values. In the **upper row** entrainment levels are shown as a function of electric field values. For both 10 (A) and 40 (B) Hz stimulation entrainment levels did not increase with electric field strength. In the **lower row** the y-axis shows Δ spike rate; here 10 Hz stimulation showed no significant slope while 40 Hz did.*

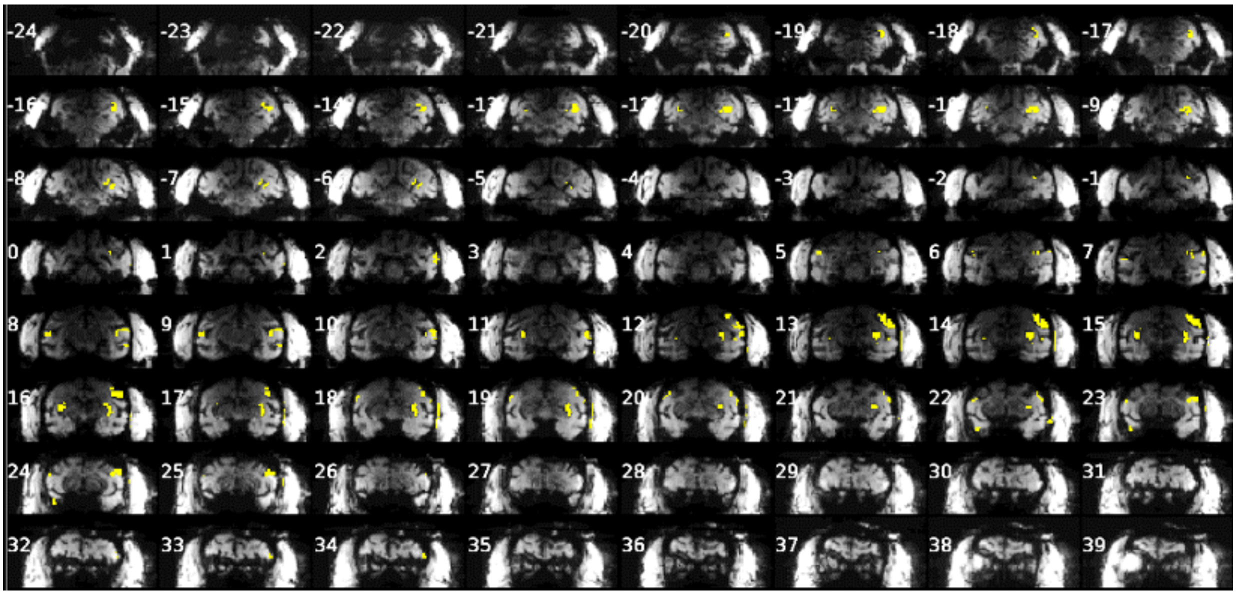

Supplementary Figure 5: Brain activation in monkey P during ECS sinewave stimulation (10 Hz, 3 mA). ECS activated target areas under the electrode as well as a network of other cortical sites.

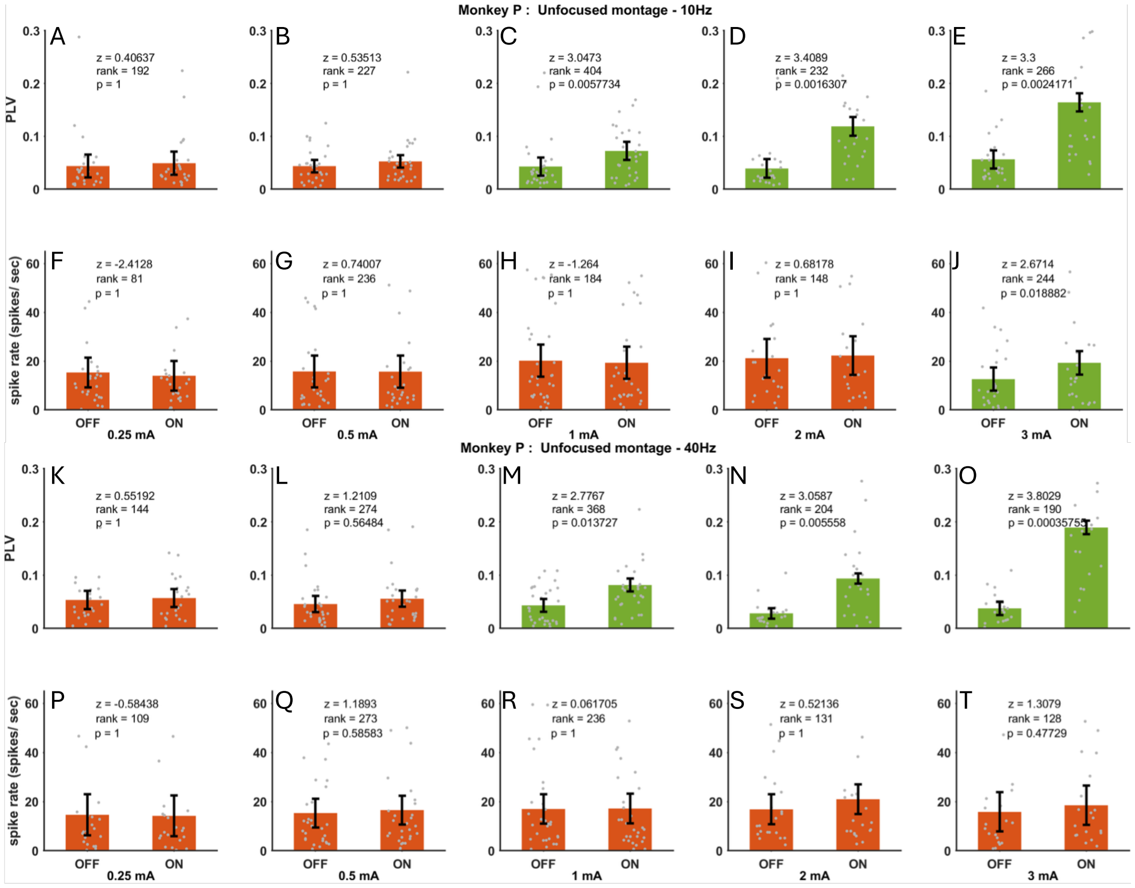

Supplementary Figure 6: Same analysis and convention as Figure 3 in the main text but only for monkey P.

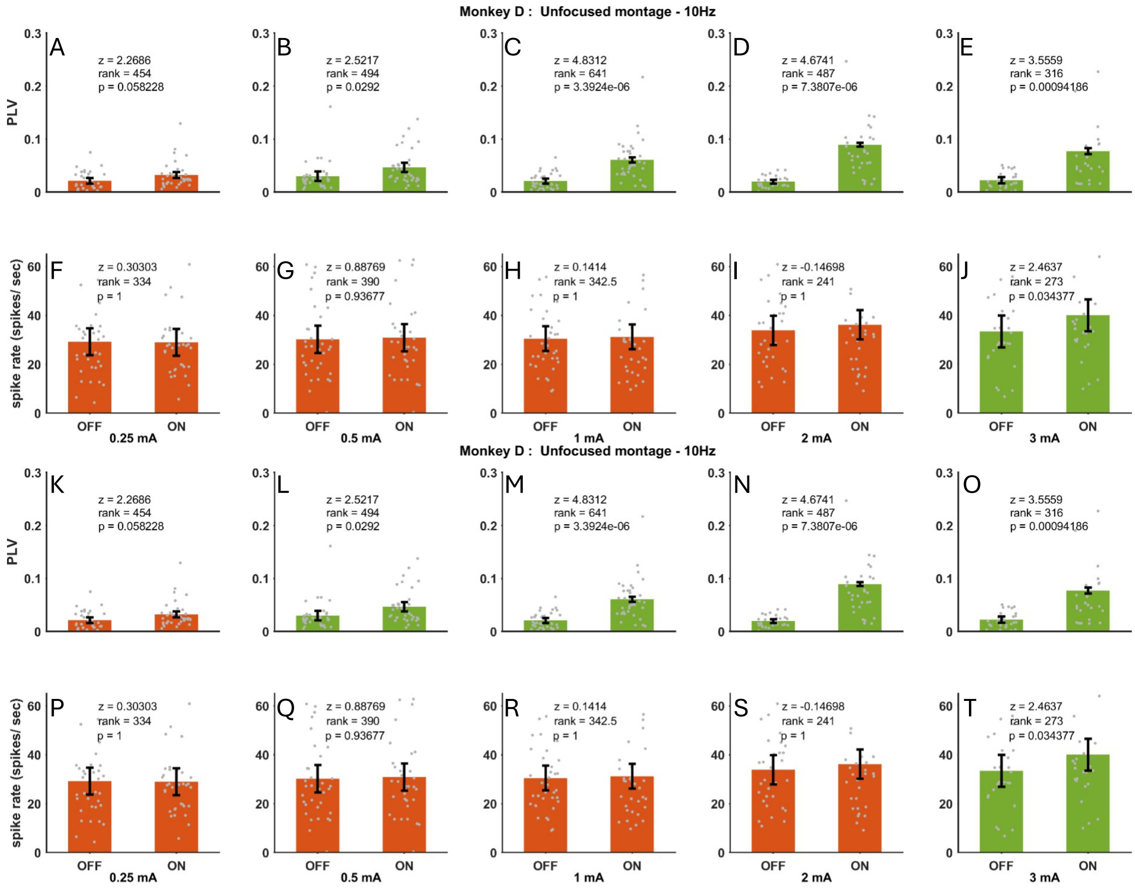

Supplementary Figure 7: Same analysis and convention as Figure 3 in the main text but only for monkey D.

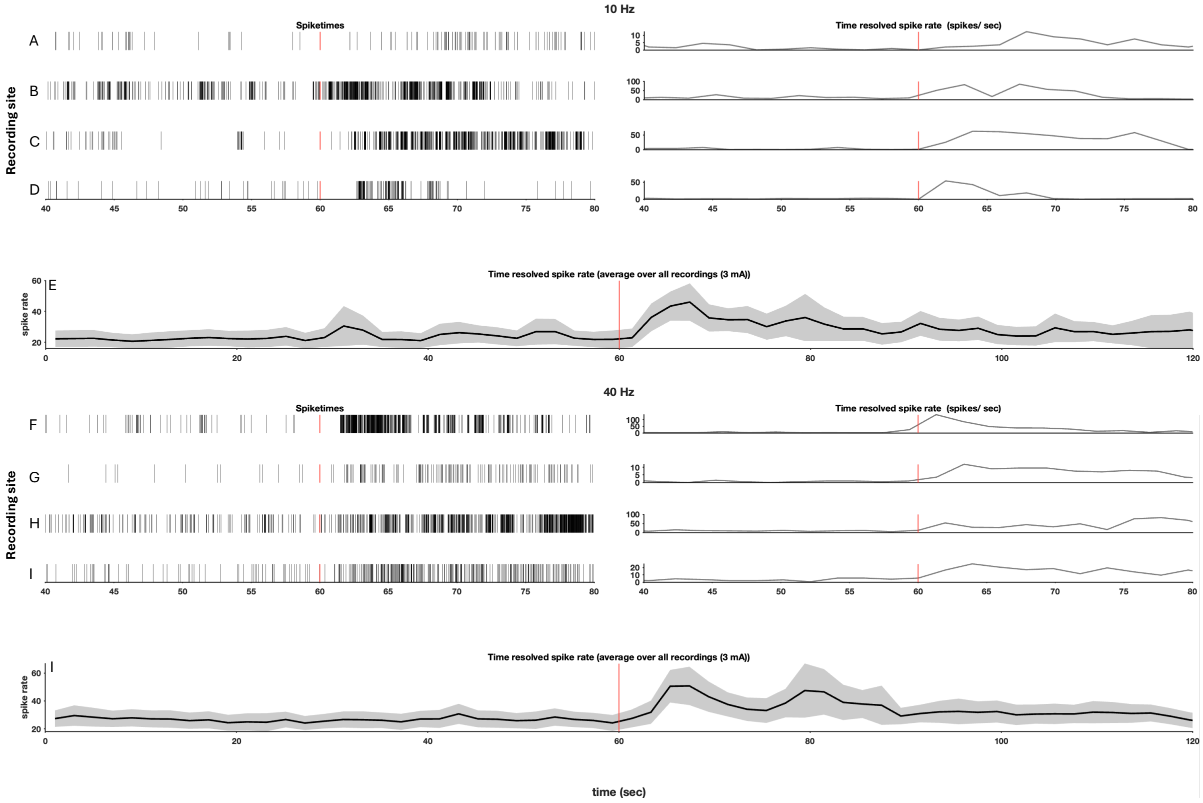

Supplementary Figure 8: Temporal development of spiking activity before and during stimulation. (A-D) Randomly selected examples of 10 Hz stimulation recordings that showed clear increase in spikjng after stimulation onset. On the left are spiketimes (zoomed in on 20 seconds before and 20 seconds after stimulation onset (red vertical line)). The right shows temporally resolved spike rate performed using a sliding window (window size is 2 seconds) over the spiking activity. (E) Average temporally resolved spike rate over all 10 Hz recordings at 3 mA. Grey shaded area depicts 95% confidence interval. (F - I) same as A-D but for 40 Hz stimulation recordings at 3 mA.

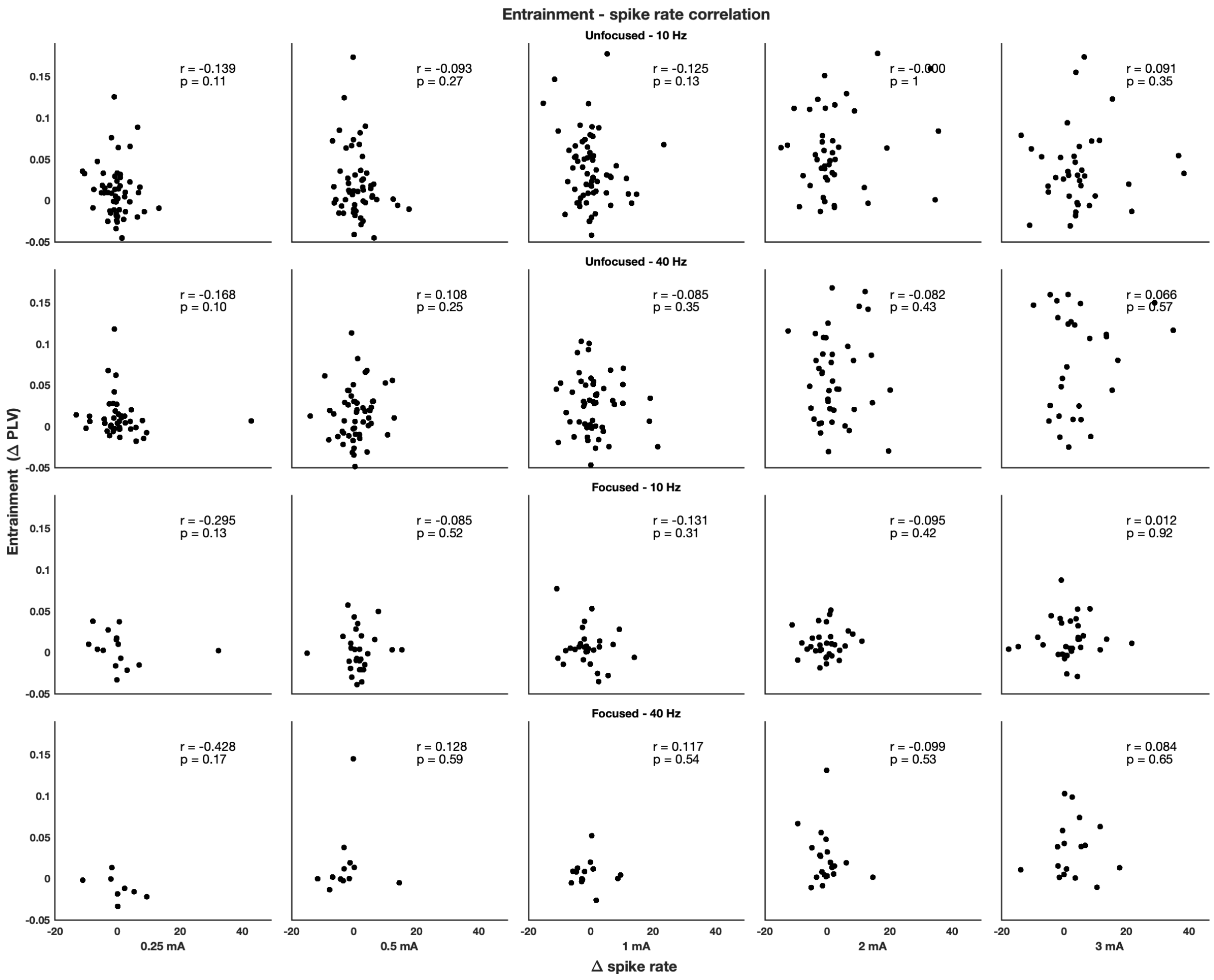

Supplementary Figure 9: Correlation between spike rate changes (x-axis) and entrainment (y-axis). There is no correlation between the two metrics.
